# Sexual dimorphism of gut microbiota at different pubertal status

**DOI:** 10.1186/s12934-020-01412-2

**Published:** 2020-07-28

**Authors:** Xin Yuan, Ruimin Chen, Ying Zhang, Xiangquan Lin, Xiaohong Yang

**Affiliations:** Department of Endocrinology, Fuzhou Children’s Hospital of Fujian Medical University, NO. 145, 817 Middle Road, Fuzhou, 350005 China

**Keywords:** Gut microbiota, Gender, Puberty, Children

## Abstract

**Background:**

Accumulating evidence infer that gut microbiome-host relations are key mediators or modulators driving the observed sexual dimorphism in some disease onset and progression. To date, the sex-differences of gut microbiota at different pubertal status have not been reported.

**Objective:**

To determine the characteristics of gut microbiota of both genders at different pubertal status.

**Methods:**

Gut microbiota was analyzed in 89 Chinese participants aged 5–15 years. Participants were divided into pre-puberty and puberty groups for both male and female. The composition of gut microbiota was investigated by 16S rRNA-based metagenomics. Ecological representations of microbial communities were computed. The prediction of metagenomic functional content from 16S rRNA gene surveys was conducted.

**Results:**

There were 49 males (9.76 ± 2.15 years) and 40 females (9.74 ± 1.63 years); 21 males and 26 females were at puberty. At genus level, *Alistipes*, *Megamonas,* *Oscillospira* and *Parabacteroides* were more prevalent in girls than in boys (*p *< 0.05). There were no significantly differences of alpha-diversity between genders, which was independent of pubertal status. The beta-diversity was significantly different in pubertal subjects between genders. Using statistical analyses, we assigned genera *Dorea*, *Megamonas, Bilophila, Parabacteroides* and *Phascolarctobacterium* as microbial markers for pubertal subjects. The predicted metabolic profiles differ in both pubertal and pre-pubertal groups between genders.

**Conclusion:**

This cross-sectional study revealed that sex differences in the gut microbiota composition and predicted metabolic profiles exist before puberty, which become more significant at puberty. The identification of novel puberty bacterial markers may disclose a potential effects of gender-related microbiota profiles on puberty onset.

## Introduction

Although the importance of the gut microbiota to human health has been of interest over several decades, few studies have addressed the effects of sex on the gut microbiota in the human intestine [1]. Recent studies using advanced techniques and large cohorts have provided detailed description of the sex differences in gut microbiota. Initially, 16S rRNA gene sequencing studies did not identify significant sex-dependent differences in the gut microbiota [2], In 2014, analysis of 16S rRNA gene sequence data set from the Human Microbiome Project (HMP) Consortium found that sex was associated with the community types identified in the stool, where males were three times more likely to have community type D in which fewer *Bacteroides* and more prevalent *Prevotella* were observed [3]. However, the conclusions of each study regarding the differences in microbial taxa between sexes are inconsistent [1]. Other large cohort studies with two independent, extensively phenotyped cohorts have been published: the Belgian Flemish Gut Flora Project (n = 1106) and the Dutch LifeLines-DEEP study (n = 1135). There was an influence of sex on the taxonomic composition of gut microbes, namely, as compared to men, women had a greater alpha diversity of the gut microbiota. In addition, men also had a lower abundance of *Clostridia, Methanobrevibacter*, and *Desulfovibrio* [4]. In general, the composition of the gut microbiota seems to be different between sexes, especially the alpha-diversity (i.e., Chao and Shannon) appears to be greater in females [5].

Given that there are gender differences in the gut microbiota, the timing of gender differences is an issue worth exploring. In an investigation of American twins, no significant differences in microbiota was found among infant twin pairs of the same sex, not the case in those of the opposite sex. However, there was greater fecal microbiota dissimilarity in opposite sex teenage (13–17 years) twin pairs compared to same sex pairs, with the inference that microbiota sex differences exist in older individuals [6]. In pre-obese diabetic (NOD) mice, the microbiota was not different between genders, however, after puberty male mice had a significantly less diverse microbiota. The abundance of *Porphyromonadaceae, Veillonellaceae, Kineosporiaceae, Peptococcaceae, Enterobacteriaceae, Lactobacillaceae, Cytophagaceae, Peptostreptococcaceae*, and *Bacteroidaceae* at the family level was more prevalent in the male mice than in the female mice [7]. Based on these observations, it is proposed that following the initial colonization, the gut microbiota stabiles at infancy, which after puberty, these emerges gender-dependent differences in gut microbial composition and function [8].

Accordingly, we hypothesized that there might be sex-different gut microbial communities in puberty compared with what is observed in pre-puberty children. To date, no study has examined the temporal change of sexual dimorphism in gut microbiota in humans spanning the dynamic hormonal changes from pre-puberty to puberty. Elucidating such relationship is important because of the known association between gut microbiota during growth and the risks of disease in adulthood [9]. Accumulating evidence implicate the role of gut microbiota in cardiovascular disease risk [10], neurobiology of mental disorders [11], immunity and disease susceptibility [12] based on gender. However, to date, the complex interaction between gender and gut microbiota communication have been insufficiently addressed. To that end we utilized 16 s rRNA gene sequencing to compare fecal microbiota communities from young children, pre-puberty to that of an adolescent age group, ranging from 5 to 15 years, and to examine when sex differences in gut microbiota become apparent.

## Methods

### Study population

This study was reviewed and approved by the Ethics Committee of Fuzhou Children’s Hospital of Fujian Medical University, and was conducted in agreement with the Declaration of Helsinki Principles. Written informed consent was obtained from all participants.

The cross-sectional study consisted of participants managed by Fuzhou Children’s Hospital of Fujian from September 2017 to March 2018. This study was limited to participants who met the following criteria: (a) ages between 5 to 15 years old, and (b) residence of Fujian province.

The exclusion criteria were as follows: Individual with any endocrine disease associated with precocious puberty, a history of antibiotic therapy, hospitalization (> 24 h) any time point during 6 months prior to the study, any gastrointestinal or chronic illness, or diarrheal disease (World Health Organization definition) during 1 month prior to the study or gastro-intestinal-related medication (antibiotics prescription).

### Dietary assessment

All participants were asked to complete a semi-quantitative food frequency questionnaire, which was developed according to the dietary habits of South China. Although participants are requested to answer the questionnaire themselves in principle, if self-completion is difficult, they are advised to seek assistance from the person who usually prepares their meals. The questionnaire assesses dietary habits during the preceding month, and consists of the following four sections: (i) daily intake and type of food, and non-alcoholic beverage items; (ii) intake frequency; (iii) usual cooking methods; and (iv) general dietary behavior. Most food and beverage items were selected from the food list of the Dietary guidelines for Chinese residents (2016).

### Clinical assessment

Height and weight were measured by trained nurses. BMI-Z scores were calculated based on Chinese reference values [13]. Tanner stage of pubertal development was assessed in all subjects by a professionally trained pediatric endocrinologist. Participants were divided into pre-puberty and puberty groups for both genders. After 12 h of fasting, 5 ml venous blood was drawn from the left arm of the participants by registered nurses. All blood samples were stored at − 80 °C and analyzed within 2 weeks of sampling. Levels of estradiol (E2) and testosterone (T) were measured by chemiluminescent immunoassays (IMMULITE 2000, Siemens Healthcare Diagnostics Products Limited, Germany) using specific reagents.

### Brief medical history

A brief medical history was obtained by questionnaire. A standardized survey was completed by all patients or parents including demographic data (birth, sex, body size and weight, and dietary habits (high-carbohydrate or high-protein diet), and pre-existing illnesses (including fever in the last 7 days). No participants smoked.

### Fecal sample collection and processing

Participants collected fecal samples at home in standard stool collection tubes. The samples were shipped immediately (within 2 h) at room temperature and were stored at − 80 °C until processing. Shipping time was usually less than 2 h.

### Genomic DNA extraction

The microbial community DNA was extracted using MagPure Stool DNA KF kit B (Magen, China) following the manufacturer’s instructions. DNA was quantified with a Qubit Fluorometer by Qubit^®^ dsDNA BR Assay kit (Invitrogen, USA) and the quality was assessed by running an aliquot on 1% agarose gel.

### Library construction

Variable regions V3-V4 of bacterial 16 s rRNA gene was amplified with degenerate PCR primers, 341F(5′-ACTCCTACGGGAGGCAGCAG-3′) and 806R(5′-GGACTACHVGGGTWTCTAAT-3′). Both forward and reverse primers were tagged with Illumina adapter, pad and linker sequences. PCR enrichment was performed in a 50 μL reaction containing 30 ng template, fusion PCR primer and PCR master mix. PCR cycling conditions were as follows: 94 °C for 3 min, 30 cycles of 94 °C for 30 s, 56 °C for 45 s, 72 °C for 45 s and final extension for 10 min at 72 °C for 10 min. The PCR products were purified with AmpureXP beads and eluted in Elution buffer. Libraries were qualified by the Agilent 2100 bioanalyzer (Agilent, USA). The validated libraries were used for sequencing on Illumina MiSeq platform (BGI, Shenzhen, China) following the standard pipeline of Illumina, and generating 2 × 300 bp paired-end reads.

The raw data were filtered to eliminate adapter contamination and low quality reads, then paired-end reads with overlap were merged to tags. And tags were clustered to OTU at 99% sequence similarity. Taxonomic ranks were assigned to OTU representative sequence using Qiime2-feature-Classifier. The database for OTUs matching was GreenGenes (v 13.8). The alpha and beta diversities, along with species screening, were analyzed based on OTU and taxonomic ranks.

### Statistical analysis

Statistical analyses of clinical data were performed using the Statistical Package for the Social Sciences software version 23.0 (SPSS Inc. Chicago, IL, USA). The normality of the data was tested using the Kolmogorov–Smirnov test. Data are expressed as mean ± SD. Comparisons of the results were assessed using independent samples t test, Mann–Whitney U test and Kruskal–Wallis test, depending on the data distribution. Comparison of rates between two groups used Chi square test. A value of *P *< 0.05 was considered statistically significant.

Statistical analysis of 16 s rDNA sequencing data was performed on alpha- (reflecting intra-individual bacterial diversity) and beta- (inter-individual dissimilarity) diversity measurements. Alpha-diversity indices contained the Shannon diversity index (calculates richness and diversity using a natural logarithm), observed OTUs, Faith’s Phylogenetic Diversity (Measures of biodiversity that incorporates phylogenetic difference between species) and Pielou’s evenness (Measure of relative evenness of species richness). Beta-diversity indices contained Jaccard distance, Bray–Curtis distance, unweighted Unifrac and weighted Unifrac using PERMANOVA methods. Kruskal–Wallis Test is used for two groups comparison. Alpha- and Beta-diversity analysis was accomplished by software QIIME2(v2019.7) [14]. Linear discriminant analysis (LDA) Effect Size (LEfSe) Analysis utilized by software LEFSE [15]. To predict metagenome functional content from 16S rRNA gene surveys, Picrust2 bioinformatics software was accessed [16] and to generate the differential pathways, the KEGG (Kyoto Encyclopedia of Genes and Genomes) pathways and STAMP [17] were used.

## Results

### Study participants

The age of the 89 participates ranged from 5.5 to 14.3 years, with a mean of 9.75 ± 1.92 years. There were 49 males (9.76 ± 2.15 years) and 40 females (9.74 ± 1.63 years). The majority (73.03%) were obese by BMI criteria (Z score of 2.08 ± 1.51). There was no significantly differences in BMI-Z between genders (p = 0.437).

Based on puberty status, the subjects were divided into pre-puberty (n = 42) and puberty groups (n = 47). There were 21 male subjects in the puberty group and 28 male subjects in the pre-puberty group, respectively. In the pre-puberty group, there was no significant difference in the level of E2 and T between males and females (p > 0.05). In the puberty group, the level of T in males was significantly higher than the females (p = 0.018). There was no statistical difference in BMI-Z scores or diet habits between genders in both the pre-puberty and puberty groups (all *p *> 0.05, Table 1 and Additional file 1: Table S1).

**Table 1:** Clinical characteristics of the study population divided by puberty status and gender

	Pre-puberty (n = 42)			Puberty (n = 47)		
	Male	Female	P value	Male	Female	P value
N	28	14		21	26	
Age (years)	8.53 ± 1.82	8.02 ± 1.22	0.346	11.38 ± 1.31	10.67 ± 0.89	0.033
Height (cm)	134.31 ± 11.63	130.19 ± 7.92	0.241	152.87 ± 7.46	145.80 ± 7.14	0.002
Weight (kg)	41.54 ± 17.75	36.09 ± 9.23	0.198	64.47 ± 9.04	45.72 ± 10.49	< 0.001
BMI (kg/cm^2^)	22.04 ± 6.31	21.03 ± 3.78	0.524	27.46 ± 2.03	21.33 ± 3.86	< 0.001
BMI-Z	1.71 ± 1.91	2.34 ± 1.49	0.249	2.57 ± 0.39	1.56 ± 1.32	0.001
Estradiol (pg/ml)	< 5	< 5	1.000	11.07 ± 8.49	33.68 ± 35.80	0.099
Testosterone (ng/dl)	5.10 ± 4.16	9.40 ± 9.76	0.445	83.20 ± 98.55	13.99 ± 20.87	0.018

### Microbiota profiles in all male and female subjects

A total of 1,005,536 sequencing reads were obtained from 89 fecal samples, with an average value of 11,289 counts per sample. We identified 150 OTUs, among which 136 OTU with ≥ 2 counts, and they were grouped in 9 phylum and 40 families.

#### Abundance profiling between genders

**Fig. 1:**
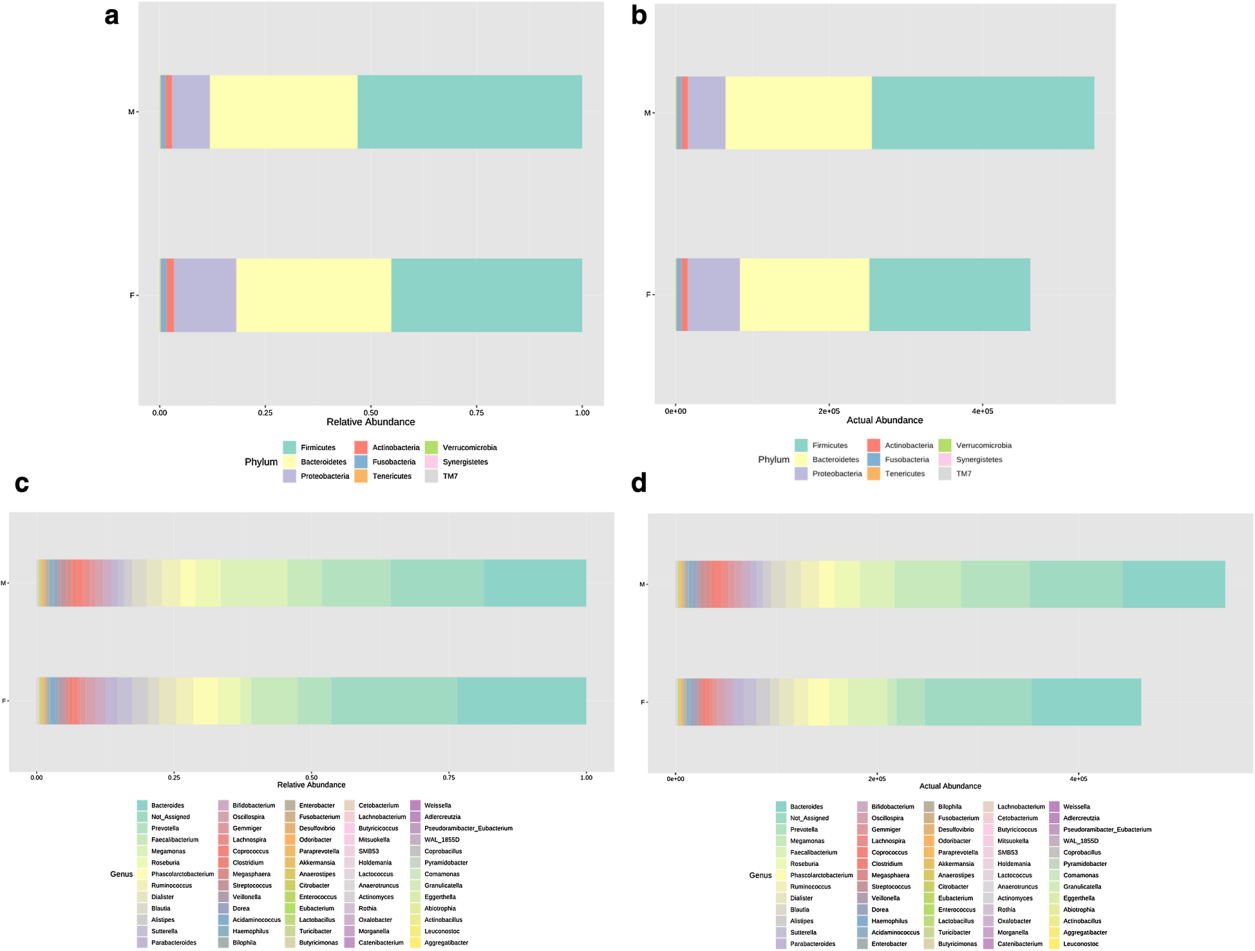
Bar chart representing Mann–Whitney U-test results on operational taxonomic units (OTUs) grouped in phyla (**a**, **b**) and in genus (**c**, **d**) of the male and female groups. Each column in the plot represents a group, and each color in the column represents: **a**, **c** the percentage of relative abundance for each OTU; **b**, **d** the values of relative abundance for each OTU

Grouping OTUs at phylum level, by Mann–Whitney U test, the relative abundances of phylum *TM7* was more prevalent in male subjects compared to female subjects (Fig. 1).

On OTUs at the genera level, by Mann–Whitney *U*-test, including all the genera (merging small taxa with counts < 10), we identified that genera *Actinomyces*, *Anaerotruncus*, *Megamonas* and *Oscillospira* were more prevalent in male subjects versus female subjects, and *Acidaminococcus, Alistipes, Bilophila*, and *Parabacteroides* were more prevalent in female subjects (all *p *< 0.05; Table 2).

**Table 2:** The mean relative abundance of gut microbiota in both genders with significantly differences at genus level

	Male	Female	Z	P value
Acidaminococcus	0.001	0.009	− 2.049	0.041
Actinomyces	0.001	0.000	− 2.030	0.042
Alistipes	0.029	0.030	− 1.978	0.048
Anaerotruncus	0.000	0.000	− 2.256	0.024
Bilophila	0.003	0.005	− 2.406	0.016
Megamonas	0.208	0.019	− 3.311	0.001
Oscillospira	0.019	0.019	− 2.252	0.024
Parabacteroides	0.016	0.023	− 2.792	0.005

#### Alpha- and beta-diversity between genders

To assess the overall differences of microbial community structures in male and female subjects, we measured ecological parameters based on alpha-diversity (Shannon, Observed OTUs, Faith’s phylogenetic diversity, and Pielou’s evenness indexes). There were no significantly differences of alpha-diversity between genders (all *p *> 0.05, Additional file 1: Table S2).

**Fig. 2:**
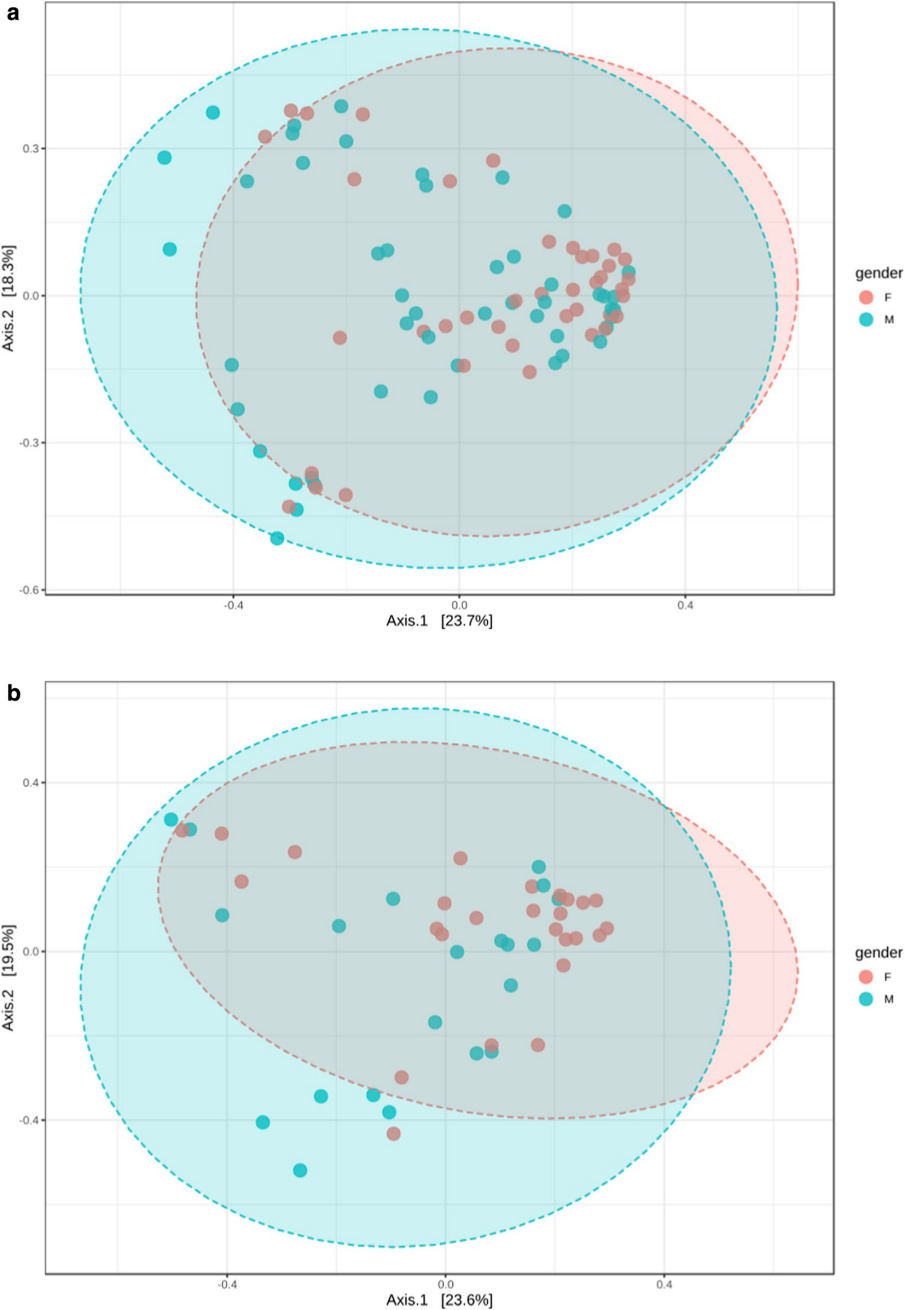
Principal coordinates analysis (PCoA) plot of male and female subjects (**a**), and of pubertal male, and pubertal female subjects (**b**). The plots show the first two principal coordinates (axes) for PCoA using Bray–Curtis Distance method

To determine the difference between microbial communities in male and female subjects, we calculated β-diversity. By Distance method Bray–Curtis, PCoA analysis, the gut microbiota samples from the male group were clustered together and separated partly from the female group: gender accounted for 23.7% of the variance in microbiota composition (*p *= 0.017, Fig. 2, Additional file 1: Table S3).

#### Bacterial taxa differences between genders

**Fig. 3:**
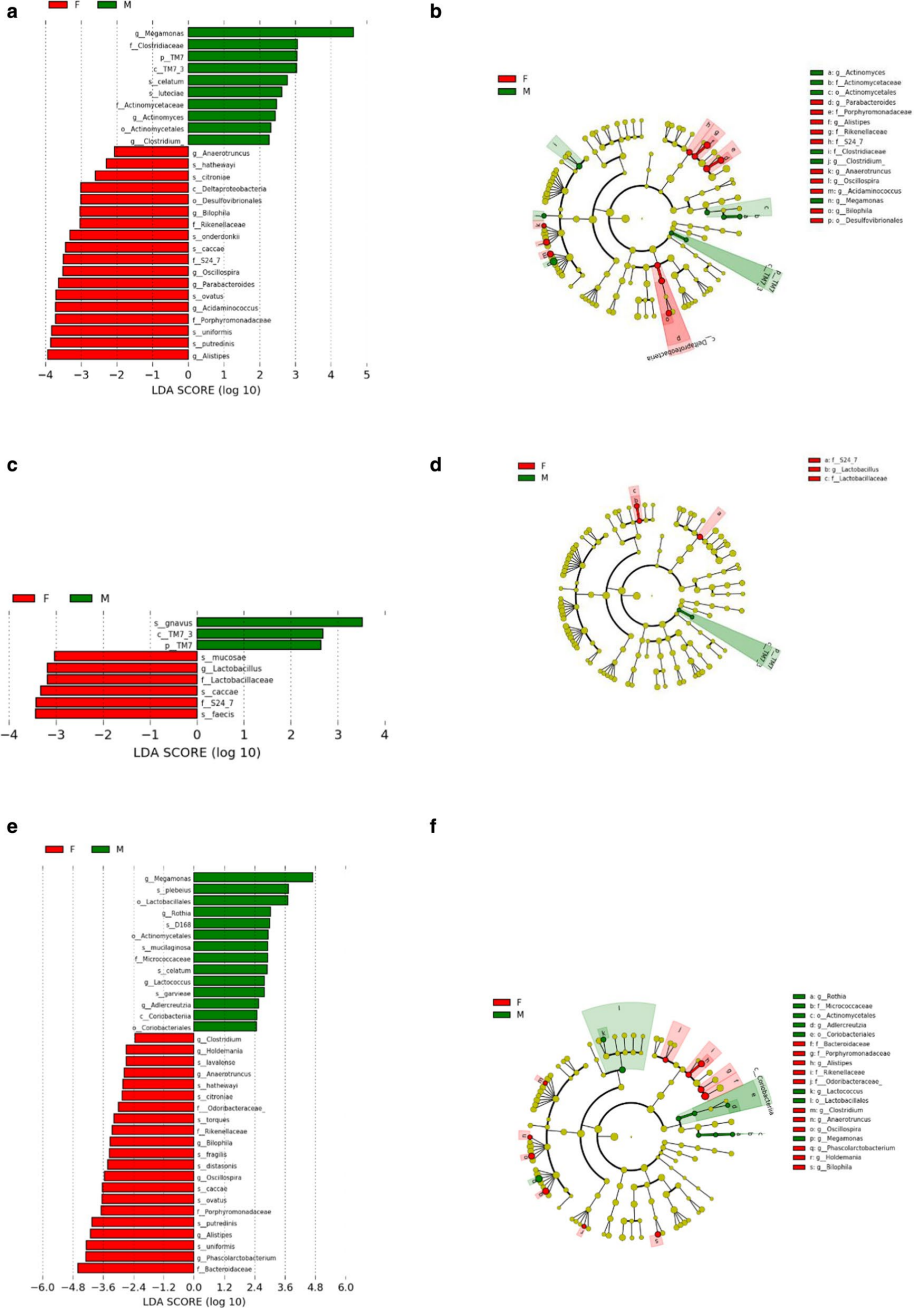
Differential biomarkers associated with genders in general population (**a**, **b**), pubertal subjects (**c**, **d**) and pre-pubertal subjects (**e**, **f**). A linear discriminant effect size (LeFse) analysis have been performed (α value = 0.05, logarithmic LDA score threshold = 2.0)

We next used LEfSe analysis to identify bacteria where the relative abundance was significantly increased or decreased in each phenotypic category. Male subjects had members of the class *TM7_3*, order *Actinomycetales*, family *Clostridiaceae, Actinomycetaceae,* genus *Meagamonas*, *Actinomyces, Clostridium*, that were significantly higher than female subjects. Meanwhile, the female subjects had members of the class *Deltaproteobacteria*, family *Rikenelaceae, S24_7, Porphyromonadaceae*, genus *Anaerotruncus, Bilophila, Oscillospira, Parabacteroides, Acidaminococcu*s that were significantly more prevalent than the male subjects (all *p *< 0.05, Fig. 3).

### Microbiota profiles in different puberty status

#### Abundance Profiling between genders in different puberty status

Grouping OTUs at phylum level, by Mann–Whitney U test, the relative abundances of phylum *TM7* was more prevalent in male subjects compared to female subjects in the pre-pubertal population (Table 3).

**Table 3:** The relative abundance of gut microbiota in different puberty status at phylum level

	Pubertal			Pre-pubertal		
	Male	Female	P value	Male	Female	P value
Actinobacteria	0.023	0.017	0.669	0.014	0.014	0.631
Bacteroidetes	0.342	0.368	0.638	0.329	0.329	0.324
Firmicutes	0.514	0.435	0.223	0.547	0.547	0.957
Fusobacteria	0.006	0.018	0.696	0.016	0.016	0.097
Proteobacteria	0.112	0.159	0.494	0.092	0.092	0.522
Synergistetes	0.000	0.000	0.724	0.000	0.000	0.774
Tenericutes	0.002	0.002	0.104	0.001	0.001	0.231
TM7	0.000	0.000	0.417	0.000	0.000	*0.044*
Verrucomicrobia	0.000	0.002	0.054	0.002	0.002	0.393

On OTUs at genera level, by Mann–Whitney *U*-test, including all the genera (merging small taxa with counts < 10). We found in the pubertal cohort that *Megamonas,* *Dorea*, *Lactococcus* and *Rothia* were more prevalent in male subjects compared to female subjects, and *Parabacteroides, Phascolarctobacterium*, *Bilophila*, *Alistipes, Oscillospira, Anaerotruncus* and *Holdemania* were more prevalent in female subjects (all *p *< 0.05; Table 4). In the pre-pubertal population, the mean relative abundance of *Lactococcus* was more prevalent in female subjects compared to male subjects (0.0002 vs 0.0001, p = 0.041).

**Table 4:** The mean relative abundance of gut microbiota in pubertal subjects at genus level

	Male	Female	Z	P value
Phascolarctobacterium	0.016	0.059	− 3.072	0.002
Megamonas	0.115	0.016	− 2.939	0.003
Rothia	0.000	0.000	− 2.745	0.006
Bilophila	0.001	0.005	− 2.574	0.010
Dorea	0.012	0.005	− 2.461	0.014
Parabacteroides	0.009	0.019	− 2.418	0.016
Alistipes	0.006	0.031	− 2.331	0.020
Anaerotruncus	0.000	0.000	− 2.328	0.020
Oscillospira	0.010	0.019	− 2.312	0.021
Lactococcus	0.001	0.000	− 2.299	0.022
Holdemania	0.000	0.000	− 2.066	0.039

By Spearman correlation analysis, relative abundances of genera *Megamonas* were positively associated with BMI-Z (r = 0.307, p = 0.036), *Parabacteroides and Holdemania* were negatively associated with BMI-Z (r = − 0.291 and − 0.317, p = 0.048 and 0.030, respectively), and the relative abundances of other differential genera (including *Alistipes, Oscillospira, Dorea*, *Lactococcus*, *Rothia, Phascolarctobacterium*, *Bilophila* and *Anaerotruncus*) were not associated with BMI-Z (all p > 0.05).

#### Alpha- and beta-diversity between genders in different puberty status

Regarding alpha-diversity, in both puberty and pre-puberty groups, the Shannon diversity index, Observed OTUs, Faith’s phylogenetic diversity and Pielou’s evenness based on OTU distribution did not reveal any significant difference between genders (all *p *> 0.05, Additional file 1: Table S2).

Regarding beta-diversity, in the pre-pubertal group, Beta-diversity also did not differ significantly between both genders. The comparisons were not significantly different (all *p *> 0.05) after correction for multiple testing (Additional file 1: Table S3); in the pubertal group, by Distance method Bray–Curtis, PCoA analysis revealed that the gut microbiota samples from the male group clustered together and separated partly from the female group, wherein gender explained 23.6% of the variance in microbiota composition (*p *< 0.015, Fig. 1).

#### Bacterial taxa differences between genders in different puberty status

In the pre-puberty group, by LEfSe analysis, male subject harbored members of the phylum *TM7* and class *TM7_3*, that were significantly higher than female subjects. In contract, the female subjects had members of the family *Lactobacillaceae, S24_7,* and genus *Lactobacillus*, that were significantly more prevalent than the male subjects (all *p *< 0.05, Fig. 3).

In the puberty group, LEfSe analysis found that male subjects compared to females had members of the order *Lactobacillales, Actinomycetales, Coriobacteriales,* class *Coriobacteriia*, family *Micrococcaceae*, genus *Megamonas, Rothia, Lactococcus, Adlercreutzia*, that were significantly more prevalent. In female subjects, members of the family *Odoribacteraceae, Rikenellaceae, Porphyromonadaceae, Bacteroidaceae*, and genus *Clostridium, Holdemania, Anaerotruncus, Bilophila, Oscillospira* were significantly higher than the male subjects (all *p *< 0.05, Fig. 3).

By Spearman correlation analysis, the relative abundances of genera *Megamonas* were positively associated with BMI-Z (r = 0.307, p = 0.036) and *Holdemania* were negatively associated with BMI-Z (r = − 0.317, p = 0.030). Other differential genera including *Lactococcus*, *Rothia, Adlercreutzia, Clostridium, Anaerotruncus, Bilophila,* and *Oscillospira* were not associated with BMI-Z (all p > 0.05).

### Microbiota profiles based on gender

#### Alpha- and beta-diversity between different puberty status

Regarding alpha-diversity, in both male and females, the Shannon diversity index, Observed OTUs, Faith’s phylogenetic diversity and Pielou’s evenness based on OTU distribution did not reveal any significant difference between pubertal and pre-pubertal subjects (all *p *> 0.05, Additional file 1: Table S2).

Regarding beta-diversity, in both male and females, beta-diversity also did not differ significantly between pubertal and pre-pubertal subjects. None of the comparisons were significantly different (all *p *> 0.05) after correction for multiple testing (Additional file 1: Table S3).

#### Bacterial taxa differences between genders in different puberty status

In male group, by LEfSe analysis, pre-pubertal subjects had members of the phylum *Verrucomicrobia* and class *Verrucomicrobiae,* order *Verrucomicrobiales,* family *Verrucomicrobiaceae,* genus *Akkermansia, Alistipes*, that were significantly higher than pubertal subjects (all *p *< 0.05, Additional file 1: Figure S1).

The relative abundances of genera *Akkermansia* and *Alistipes* were not associated with BMI-Z (all p > 0.05) by Spearman correlation analysis.

In females, by LEfSe analysis, pre-puberty subjects had members of the order *Actinomycetales, Pasteurellales,* family *Micrococcaceae*, Pasteurellaceae, genus *Rothia, Haemophilus*, which were significantly more prevalent than pubertal subjects. Pubertal subjects had members of the genus *Phascolarctobacterium*, *Veillonella* were significantly more prevalent than the pre-pubertal subjects (all *p *< 0.05, Additional file 1: Figure S1).

### Correlations between sex hormone and bacterial abundance

To evaluate correlations between bacteria and serum sex hormones (testosterone and estradiol), Spearman’s rank analysis was adopted. In the male subjects, the abundance of genus *Clostridium* was positively associated with the level of T (r = 0.500, p = 0.021). In the female subjects, the abundance of genus *Roseburia* was positively associated with the level of E2 (r = 0.433, p = 0.0499).

### Detecting microbial biomarkers in both genders

Discriminant analysis (DA) based on univariate ANOVAs, Fisher’s coefficient and leave-one-out classification were performed to define a model based on the capability of OTUs to discriminate the four groups of study participants (pubertal male subjects, pubertal female subjects, pre-pubertal male subjects, and pre-pubertal female subjects).

**Fig. 4:**
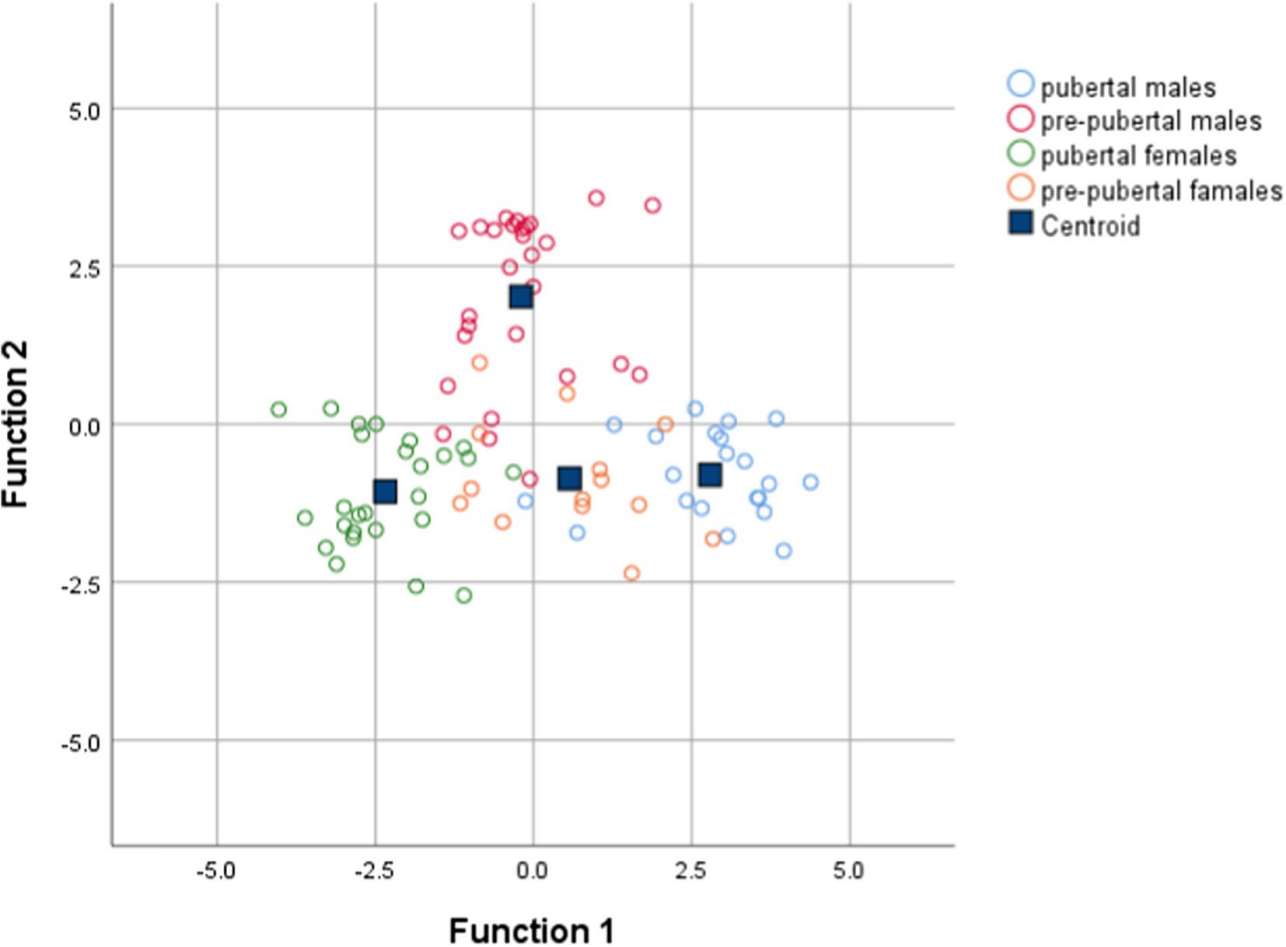
Canonical discriminant plot. Scatter plot of canonical discriminant analysis (DA) based on univariate ANOVA and Fisher’s coefficient applied to all OTUs of samples belonging to pubertal males, pubertal females, pre-pubertal males, and pre-pubertal females

By DA, 89.9% of the original grouped subjects were correctly classified, and the canonical discriminant plot revealed a clear separation between pubertal males and pubertal females (Fig. 4 and Additional file 1: Table S3). In particular, the Figure illustrates a clear separation between the samples belonging to the four groups, with most of the samples being close to the centroid of the group of belonging, although a lower separation was observed between pre-pubertal males and pre-pubertal females. However, applying a cross-validation (CV) test, we found that only 16.9% of cases were correctly classified, revealing a low capability of the entire OTUs set to discriminate the four groups (Additional file 1: Table S4).

**Fig. 5:**
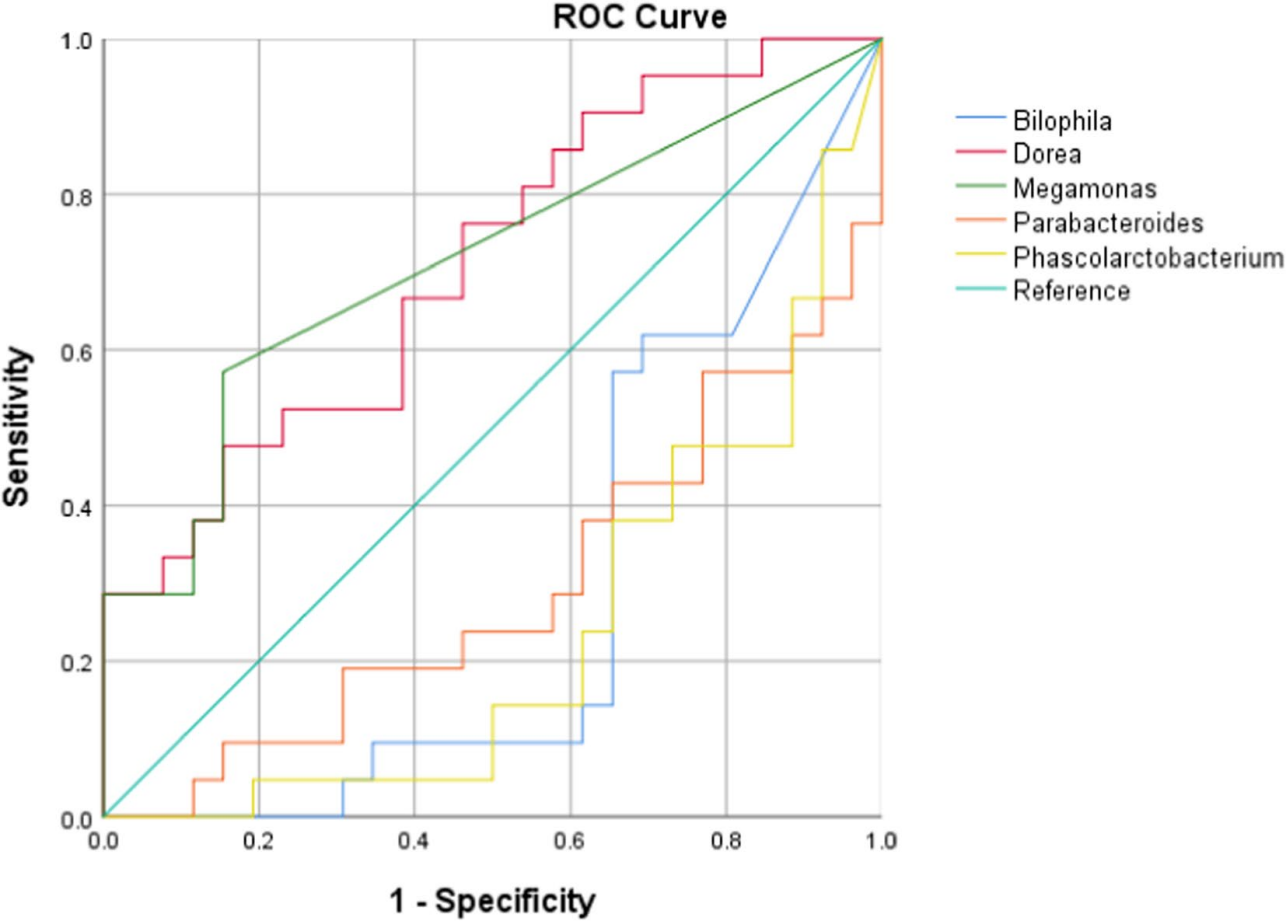
Receiver operating characteristic (ROC) curve plots. The areas under the ROC curves (AUROC) represent the specificity and sensitivity of the five OTUs (AUROC > 0.7) able to discriminate the gender in pubertal subjects

We further tested the discriminatory power of the OTUs at genus level in correctly classifying groups by applying the average area under the ROC (AUROC). In pre-puberty population, the AUROC for the pre-pubertal females was < 0.7 for all genera, revealing a low capability to discriminate genders. In the puberty cohort, the AUROC for the pubertal males was 0.711 and 0.712 for genera *Dorea* and *Megamonas,* respectively, and the AUROC for the pubertal females was 0.718, 0.707 and 0.763 for genera *Bilophila, Parabacteroides* and *Phascolarctobacterium,* respectively (Fig. 5).

For male/female population, AUROC values were all < 0.7, considered not accurate in discriminating different pubertal status.

### Metabolic pathway predictions

**Fig. 6:**
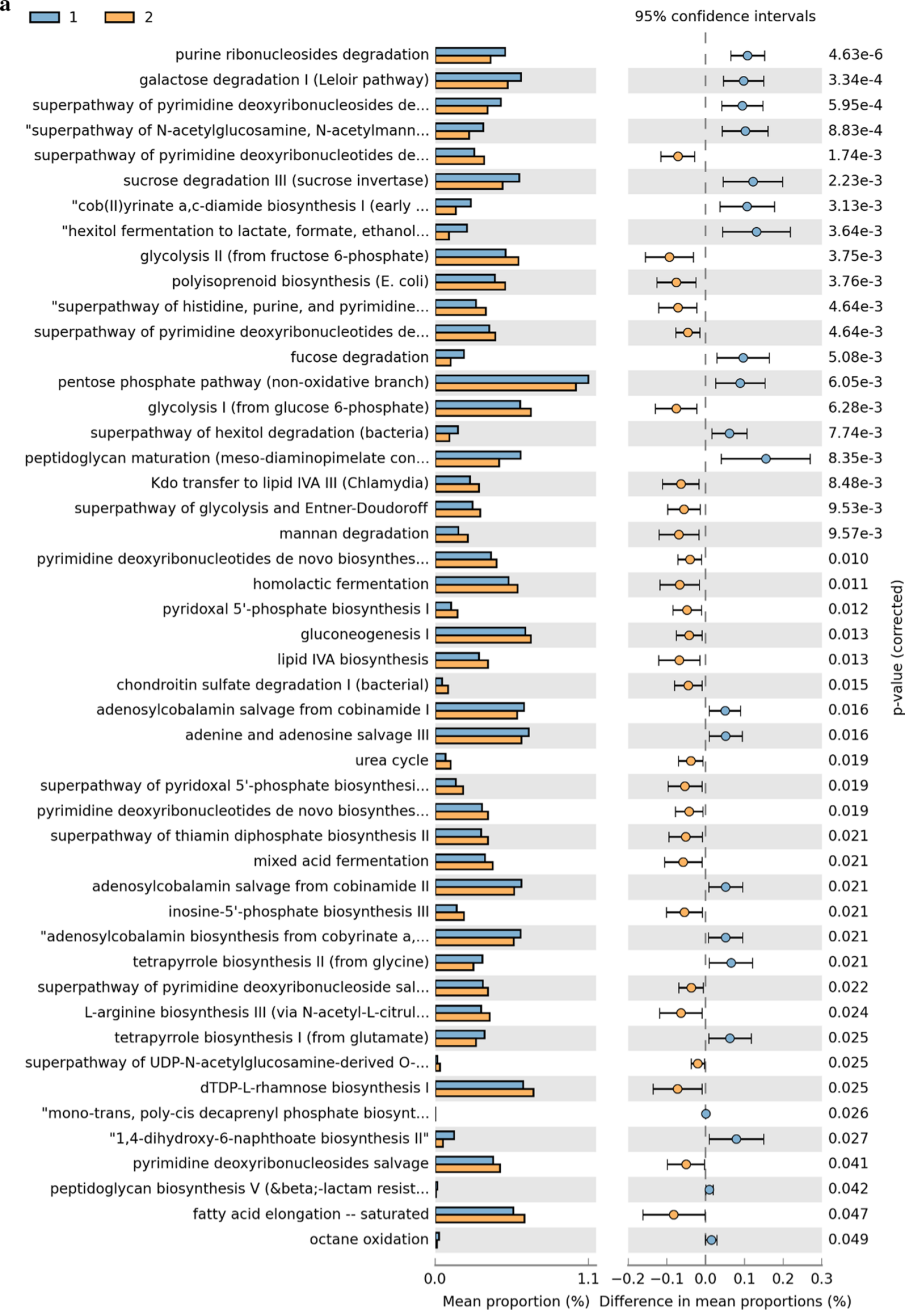

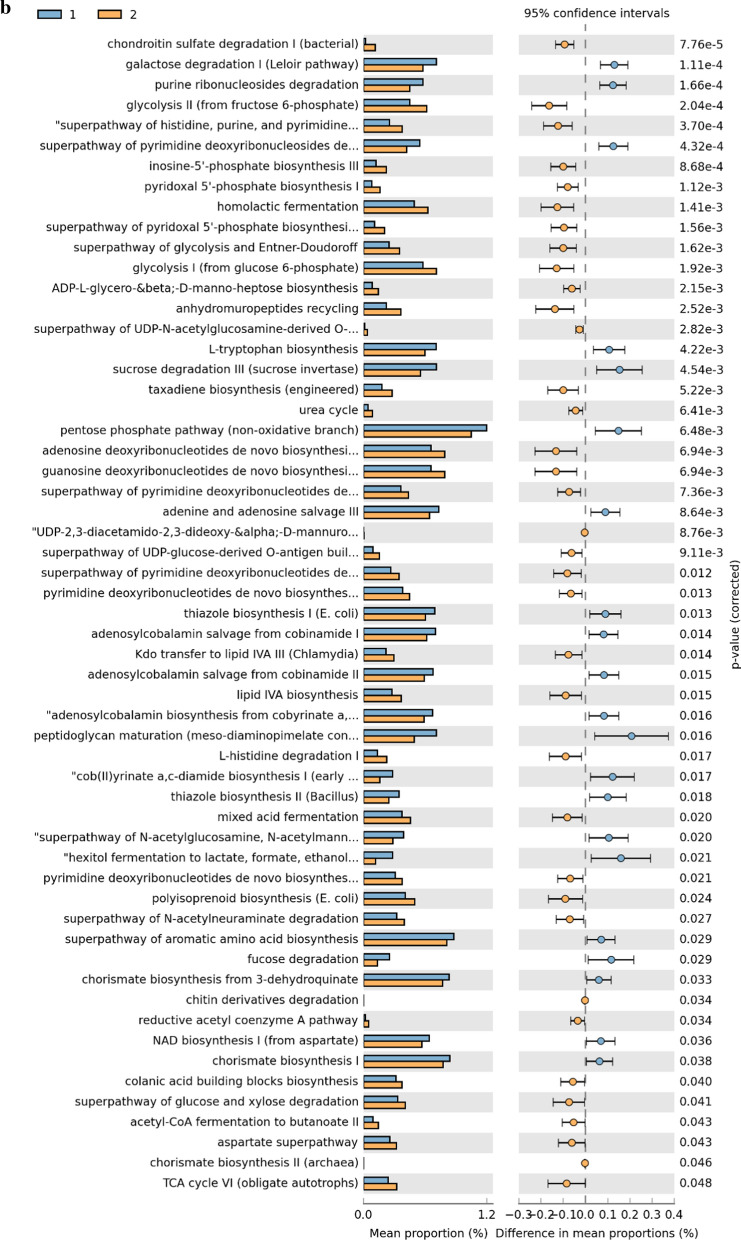

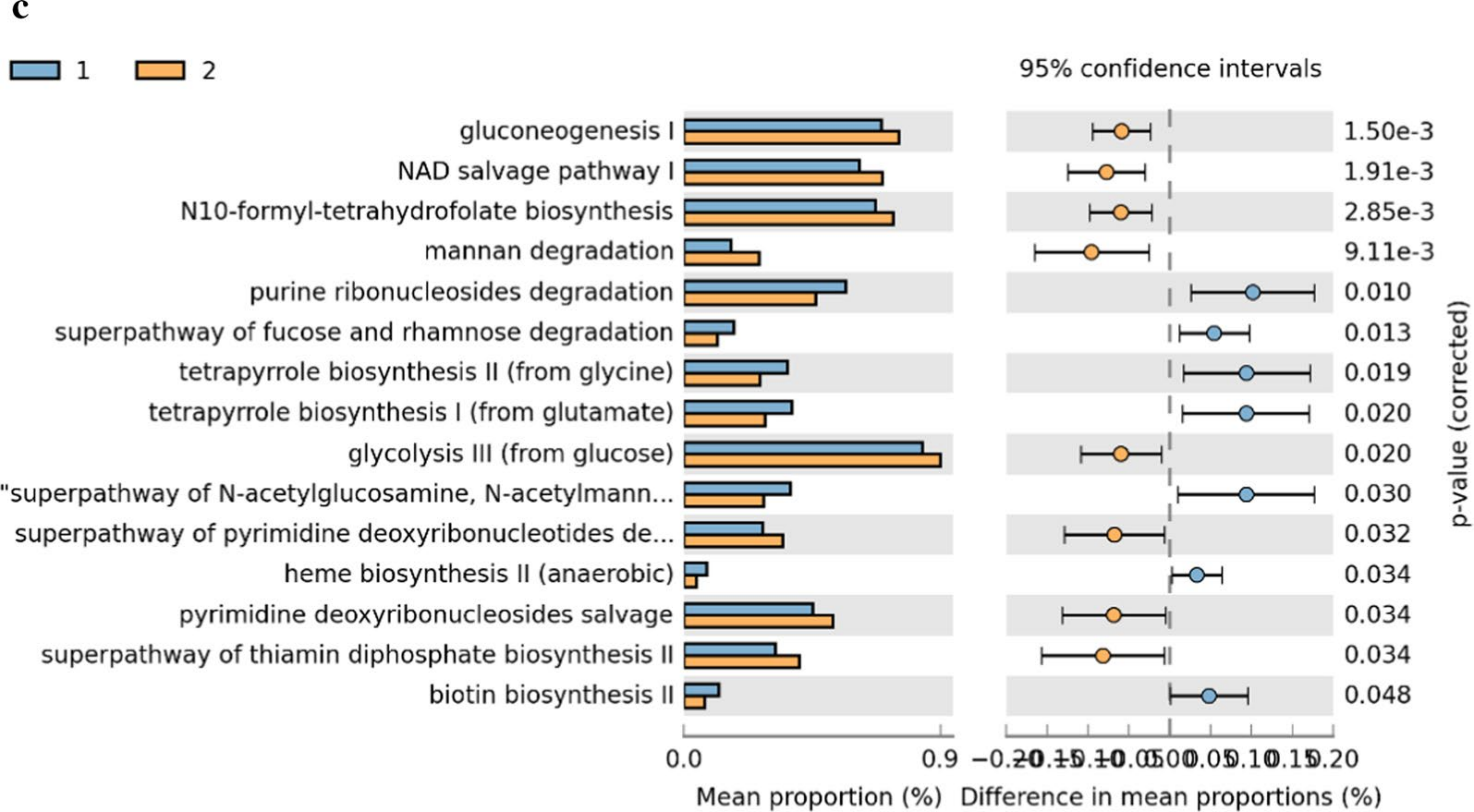
KEGGs biomarkers associated with male (1) and female (2) conditions in general population (**a**), pubertal subjects (**b**) and pre-pubertal subjects (**c**)

A total of 48 KEGG pathways were generated using the composition of the gut microbiota based on PICRUSt2 in males versus females (Fig. 6, Additional file 1: Table S5). Some carbohydrate pathway (e.g., galactose, fucose and pentose phosphate pathway), and nucleotide metabolism pathways (e.g., adenine and pyrimidine deoxyribonucleosides) were increased in male subjects. Whereas in females, some pathways associated with carbohydrate (gluconeogenesis, glycolysis), nucleotide metabolism (e.g., purine ribonucleosides and pyrimidine deoxyribonucleosides), amino acid metabolism (urea cycle) and lipid metabolism (fatty acid elongation) were increased (P < 0.05).

In the comparison between pubertal subjects, we obtained 21 differential pathways associated with males and 36 associated with females (Fig. 6, Additional file 1: Table S6). Moreover, 15 differential metabolic patterns were observed between pre-pubertal males and pre-pubertal females. Seven KEGG pathways were significantly upregulated in the pre-pubertal males group while 8 were significantly upregulated in pre-pubertal females group (Fig. 6, Additional file 1: Table S7).

## Discussion

Sex differences in the prevalence, and sometimes severely, of immunity deficiencies, most major neuropsychiatric and neurological disorders, and even cardiovascular disease are well described in literature. In addition to factors such as epigenetic, life experience, socioeconomic status, physiological state, and perceived stress levels, gut microbiome-host relations have been implicated as key mediators or modulators begetting the observed sexual dimorphism in disease onset and progression. Charactering the gut microbiota at different life stages and the timing of gender dimorphism is pertinent to the study of gender dimorphism of diseases [18]. Even though the underlying mechanism is unresolved, gender differences in the gut microbiota may contribute to the discordance in the development and presentation of various diseases [1]. In the past, some adverse drug effects that occurred preferentially in females were not apparent until widespread use in clinical practice [19]. Amorously, a sex-dependent effect may be overlooked if a microbiota-based therapeutic strategy is adopted by clinicians [1].

Data from the sequencing of bacterial 16S rRNA genes has yielded insight into possible symbiotic or baneful interactions between sex and the gut microbiota. In a large cohort study (n = 1135) in the Netherlands, sex was associated with 12 microbial species and 43 metabolic pathways, and females had a higher abundance of *Akkermansia muciniphila* even after correcting for all confounding factors, such as diet, lifestyle, and medication [20]. In Italy, the mucosa-associated microbiota in females had a higher abundance of *Actinobacteria, Lactobacillales, Streptococcaceae*, and *Bifidobacterium* and less *Veillonellaceae* and unclassified *Clostridia*. At the species level, *Gemmiger formicilis* was associated with the males and *Bifidobacterium adolescentis* with the females [21]. In our study, we found that the gut microbiota of male subjects was significantly different than female subjects, not only the beta-diversity and gut microbiota composition, but also the relative abundance in taxonomic. Furthermore, in the comparison between pubertal subjects, 21 differential pathways were associated males and 36 associated with females.

Few reports explore sex differences in gut microbiome profile during adolescence. However, in adults, alpha diversity increases slightly until middle age with greater microbiome diversity seen in females [18]. Also, de la Cuesta-Zuluaga J investigated the association of age, sex, and gut bacterial alpha diversity in three large cohorts of adults from four geographical regions: subjects from the United States and United Kingdom in the American Gut Project citizen-science initiative and two independent geographic cohorts of Colombians and Chinese. In three of the four cohorts, they found sex-dependent differences that were more pronounced in younger adults than in middle-aged adults, with women having higher alpha diversity than men. In contrast to the other three cohorts, no association of alpha diversity with age or sex was observed in the Chinese cohort [5]. The studies reporting the sex-differences of gut microbiota in Asia reached discordant conclusions from those of the Western studies. In one Japanese study, there was no significant difference in the α-diversity between males and females [22], yet found significantly higher levels of *Prevotella*, *Megamonas*, *Fusobacterium*, and *Megasphaera* in the males, and *Bifidobacterium*, *Ruminococcus*, and *Akkermansia* in females. One Chinese study reported that there were no overall significant taxonomic differences between males and females [23]. In concert with these studies, no association of alpha diversity with sex or pubertal status was found in our study, not only in the overall population, but also in the pubertal population. However, due to the different ages of the study subjects, the gut microbiota might yet corelated with developmental age in a large study group was analgged, more records, especially prospective, is warranted to assess the relationship between alpha-diversity and race, geographic location and other factors.

Animal studies, mainly in mice, have clearly shown sex-specific differences in the composition of gut microbiota [7, 24–27]. Org et al. found that when the gut microbiota of 89 different inbred mouse strains were analyzed independently, clear differences in the gut microbiota composition and diversity were observed between the sexes within each strain. In the total cohort, the phylum *Actinobacteria* and *Tenericutes* were more abundant in male than female mice [25]. In another study using two different mouse strains (BALB/c and B6), the males had a lower microbial diversity and richness than the females, and sex explained 11.6% of the variance in microbiota composition [26]. Fecal microbiota transplantation (FMT) experiment showed evidence of the effect of sex difference on shaping the gut microbiota. After transplanting the same specific pathogen-free feces from a female into germ-free (GF) mice of both sexes, the gut microbiota after puberty were distinctly segregated according to the sex of the recipient mice [7]. Similarly, a fecal suspension from a 32-year-old woman was administered to both male and female GF adult rats, whereupon the microbiota clustered according to the sex of the host animal despite identical fecal inoculum [28].

Studies in rodents demonstrated that the gut microbiota diverged after puberty in a sex-specific manner. The gut microbiota was found to be undistinguishable between male and female mice at 3 weeks of age (pre-puberty) but the α-diversity was, based on sex, dissimilar at 6 weeks of age (post-puberty) [7, 24]. Sex-differences in the mouse microbiota composition arise during puberty with the males acquiring a distinct gut microbiota composition compared to pre-pubertal mice of both sexes [29, 30]. Considering that the gut microbiota is similar prior to puberty and then diverge after puberty, it is logical to ponder whether sex hormones play a role.

In this study, we found significant differences of gut microbiota between pubertal males and pubertal females, which infer that sex steroid hormones are closely linked with changes in the gut microbiota, or, perhaps, the converse cause- and- effect relationship is ensuing. Previously reported, gut microbiota diversity increased in infancy and stabilized by 5 years of age without any apparent sex-differences per se [31, 32]. We found that the diversity was stabilized without any apparent sex-differences in pre-pubertal subjects after 5 years of age, even though the female subjects had members of the family *Lactobacillaceae, S24_7,* and genus *Lactobacillus* which were significantly higher than the male subjects, and 15 differential metabolic patterns differentially in both genders, which were further confirmed by the results of DA. We speculate that differences associated with gender in the gut microbiota could be attributable to sex chromosomes in intestinal cell, circulating or paracrine hormones.

Evidence for sex steroid activation of the gut flora comes from studies of the effects of gonadectomy on the microbiota: Oestrogens regulate gut microbiota composition since beta diversity of ovariectomised (OVX) female mice clusters with male mice. Further compositional analysis in the fecal microbiota of oestradiol treated males or OVX females showed clustering with females, separate from male or OVX female cluster, suggesting that oestrogens, modulate gut microbiota composition [33]. Several gonadectomy studies demonstrated that differences in gut microbiota composition between sexes were clearly mediated at least in part by sex hormones. Furthermore, in mice testosterone treatment after gonadectomy prevented the changes in gut microbiota composition that were apparent in untreated males [25, 34]. Because gender plays a role in the maturation of the gut microbiota at puberty, it is uncertain as to what initiates the gut microbiota shift. Our data revealed differential gut microbiota between pubertal and pre-pubertal subjects in both genders, suggesting beside the role of sex steroids, the gut microbiota is enriched at puberty through other non-hormonal influencing factors.

Finally, we further investigated the functional profiles of the gut microbial communities by using PICRUSt2 analysis. Multiple metabolic pathways (e.g. carbohydrate, amino acid, and lipid) were increased in the gut microbiome from female subjects. Plausibly, their metabolites (e.g. fatty acid) may participate in adipose tissue remodeling during puberty [35]. Furthermore, reduced E2 levels during the menopause transition have been linked to increased risk of type 2 diabetes [36]. In this study, levels of E2 impacted bacteria carbohydrate metabolic pathways, which may account for the protective metabolic effects of E2 which wane during aging [10, 37].

This cross-sectional study revealed sexual dimorphism of gut microbiota at different pubertal status. However, a longitudinal study wherein the participants are followed over the period of an extended period (pre-puberty to puberty) would confirm a dynamic change in gut microbiome before and after puberty. Notwithstanding impact of diet on the gut microbiome in this limited study, the diverse dietary habits across China could not be mirrored. Furthermore, the finite information on the dietary recall/questionnaire renders any conclusion concerning nutrient and the adolescent microbiome suspect.

## Conclusion

The present study is the first to assess the gender-differences of gut microbiota in both pubertal and pre-pubertal subjects. Our results indicate that such gut communities were sufficiently different in the pre-pubertal age group. Specifically, genera *Dorea*, *Megamonas, Bilophila, Parabacteroides* and *Phascolarctobacterium* were signed as microbial markers for pubertal subjects. The sex-dependent gut microbiota diversity is, in part, related in sex hormone.

## Supplementary information

**Additional file 1: Figure S1.** Differential biomarkers associated with puberty status in male subjects (a) and female subjects (b). A linear discriminant effect size (LeFse) analysis have been performed (α value = 0.05, logarithmic LDA score threshold = 2.0). **Table S1.** Dietary habits of the study population divided by puberty status and gender (Chi square test). **Table S2.** Comparison of alpha-diversity between different gender. **Table S3.** Comparison of beta-diversity between different gender. **Table S4.** Discriminant analysis table based on statistically different OTUs and 4 subject groups. **Table S5.** KEGGs biomarkers in males and females. **Table S6.** KEGGs biomarkers in pubertal males and pubertal females. **Table S7.** KEGGs biomarkers in pre-pubertal males and pre-pubertal females.

## Data Availability

The datasets supporting the conclusions of this article are included within the article and its additional files.
