# Supplementary material for: Sexual dimorphism of gut microbiota at different pubertal status
Source: Microb Cell Fact. 2020 Jul 28;19:152. doi: 10.1186/s12934-020-01412-2 (PMC7390191; doi:10.1186/s12934-020-01412-2)
Supplement: Supplementary file 1 — Additional file 1: Figure S1. Differential biomarkers associated with puberty status in male subjects (a) and female subjects (b). A linear discriminant effect size (LeFse) analysis have been performed (α value = 0.05, logarithmic LDA score threshold = 2.0). Table S1. Dietary habits of the study population divided by puberty status and gender (Chi square test). Table S2. Comparison of alpha-diversity between different gender. Table S3. Comparison of beta-diversity between different gender. Table S4. Discriminant analysis table based on statistically different OTUs and 4 subject groups. Table S5. KEGGs biomarkers in males and females. Table S6. KEGGs biomarkers in pubertal males and pubertal females. Table S7. KEGGs biomarkers in pre-pubertal males and pre-pubertal females. [file 12934_2020_1412_MOESM1_ESM.docx]

a:


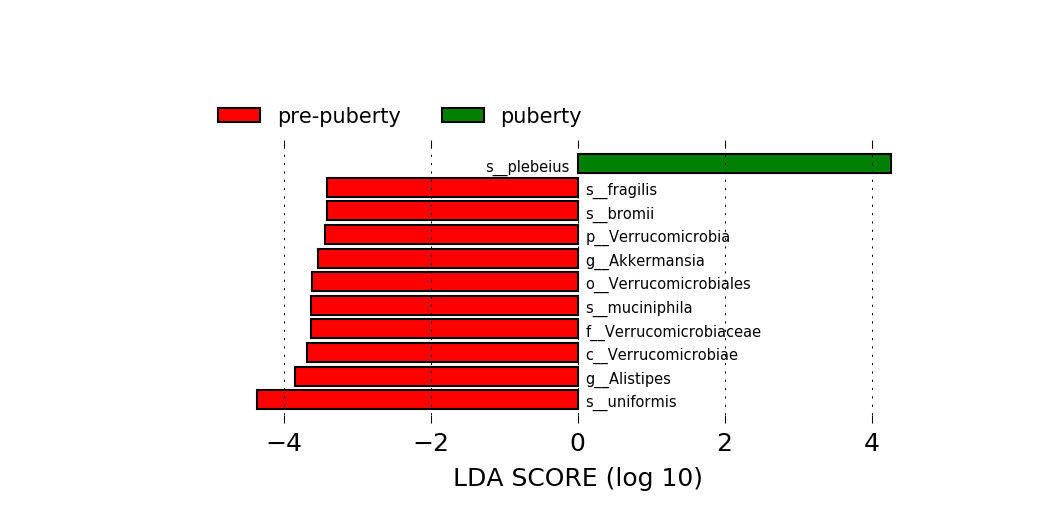


b:


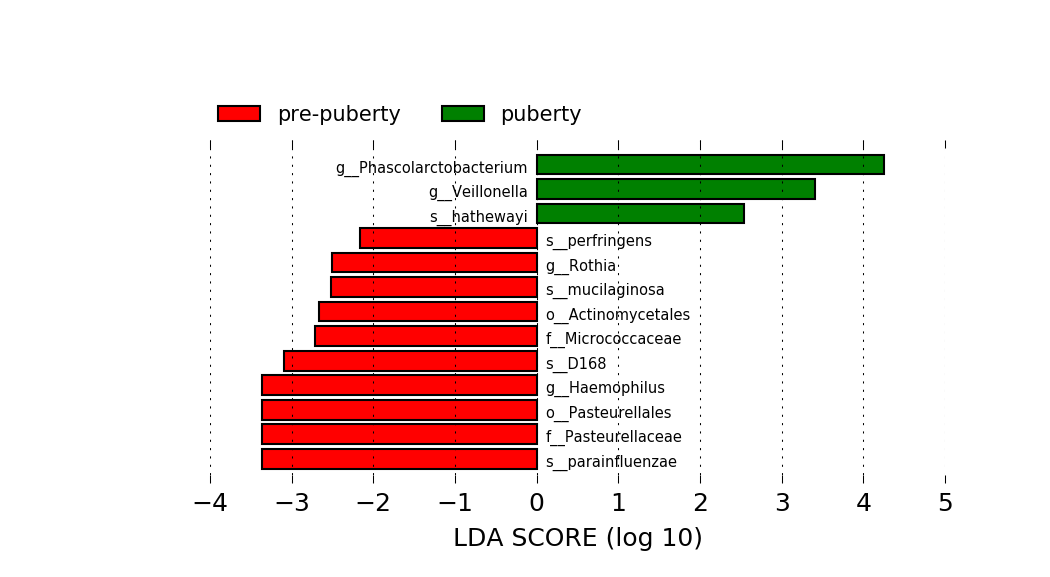


Figure S1. Differential biomarkers associated with puberty status in male subjects (a) and female subjects (b). A linear discriminant effect size (LeFse) analysis have been performed (α value = 0.05, logarithmic LDA score threshold = 2.0).

**Table S1** Dietary habits of the study population divided by puberty status and gender (Chi-square test)

|  | pre-puberty  (n=42) | | | Puberty  (n=47) | | |
| --- | --- | --- | --- | --- | --- | --- |
|  | Male  (n=28) | Female  (n=14) | P value | Male  (n=21) | Female  (n=26) | P value |
| **Daily intake and type of food** | | | | | | |
| Cereals and vegetable-based | 6 | 5 | 0.459 | 6 | 9 | 0.785 |
| Cereals and meat-based | 22 | 9 |  | 15 | 17 |  |
| **Intake frequency** | | | | | | |
| Three meals per day | 25 | 14 | 0.539 | 18 | 24 | 0.644 |
| Snacks besides meals | 3 | 0 |  | 3 | 2 |  |
| **Usual cooking methods** | | | | | | |
| Fried | 26 | 11 | 0.313 | 19 | 24 | 1.000 |
| Steamed | 2 | 3 |  | 2 | 2 |  |
| **Ingestion frequency of sugary drinks** | | | | | | |
| almost everyday | 3 | 2 | 0.855 | 0 | 1 | 0.751 |
| three times a week | 3 | 1 |  | 2 | 2 |  |
| occasionally | 11 | 4 |  | 10 | 13 |  |
| almost not | 11 | 7 |  | 9 | 10 |  |

**Table S2** Comparison of alpha-diversity between different gender

| groups | alpha-diversity index | H | P value |
| --- | --- | --- | --- |
| Male v.s. female | Shannon | 2.430 | 0.119 |
|  | Observed OTUs | 2.916 | 0.088 |
|  | Faith’s phylogenetic diversity | 2.083 | 0.149 |
|  | Pielou’s evenness | 1.258 | 0.262 |
| Pubertal males v.s. pubertal females | Shannon | 1.145 | 0.285 |
|  | Observed OTUs | 2.182 | 0.140 |
|  | Faith’s phylogenetic diversity | 2.645 | 0.104 |
|  | Pielou’s evenness | 0.202 | 0.653 |
| pre-pubertal males v.s. pre-pubertal females | Shannon | 1.256 | 0.262 |
|  | Observed OTUs | 2.695 | 0.201 |
|  | Faith’s phylogenetic diversity | 1.028 | 0.311 |
|  | Pielou’s evenness | 0.377 | 0.539 |
| pre-pubertal males v.s. pubertal males | Shannon | 2.216 | 0.642 |
|  | Observed OTUs | 1.806 | 0.179 |
|  | Faith’s phylogenetic diversity | 2.000 | 0.157 |
|  | Pielou’s evenness | 0.004 | 0.952 |
| pre-pubertal females v.s. pubertal females | Shannon | 1.421 | 0.999 |
|  | Observed OTUs | 0.798 | 0.372 |
|  | Faith’s phylogenetic diversity | 0.930 | 0.335 |
|  | Pielou’s evenness | 0.503 | 0.478 |

**Table S3** Comparison of beta-diversity between different gender

| groups | beta-diversity index | pseudo-F | P value |
| --- | --- | --- | --- |
| Males v.s. females | Bray-Curtis distance | **1.455** | **0.017** |
|  | Jaccard distance | **1.300** | **0.018** |
|  | Unweighted-unifrac | **2.302** | **0.008** |
|  | Weighted-unifrac | 1.849 | 0.104 |
| puberty males v.s. pubertal females | Bray-Curtis distance | **1.612** | **0.015** |
|  | Jaccard diatance | **1.507** | **0.009** |
|  | Unweighted-unifrac | **2.427** | **0.015** |
|  | Weighted-unifrac | 1.795 | 0.09 |
| pre-pubertal males v.s. pre-pubertal females | Bray-Curtis distance | 1.148 | 0.212 |
|  | Jaccard diatance | 1.063 | 0.256 |
|  | Unweighted-unifrac | 1.376 | 0.115 |
|  | Weighted-unifrac | 1.260 | 0.243 |
| Pre-puberty males v.s. pubertal males | Bray-Curtis distance | 1.188 | 0.168 |
|  | Jaccard distance | 1.111 | 0.096 |
|  | Unweighted-unifrac | 1.304 | 0.121 |
|  | Weighted-unifrac | 0.513 | 0.696 |
| Pre-puberty females v.s. pubertal females | Bray-Curtis distance | 1.167 | 0.199 |
|  | Jaccard diatance | 1.228 | 0.065 |
|  | Unweighted-unifrac | 1.086 | 0.310 |
|  | Weighted-unifrac | 1.305 | 0.231 |

**Table S4.** Discriminant analysis table based on statistically different OTUs and 4 subject groups

|  |  | group | **predicted group membership** | | | |  |
| --- | --- | --- | --- | --- | --- | --- | --- |
|  |  |  | pubertal males | pre-pubertal males | pubertal females | pre-pubertal females | total |
| **Original** | count | pubertal males | 20 | 0 | 0 | 1 | 21 |
|  |  | pre-pubertal males | 1 | 22 | 4 | 1 | 28 |
|  |  | pubertal females | 0 | 0 | 26 | 0 | 26 |
|  |  | pre-pubertal females | 2 | 0 | 0 | 12 | 14 |
|  | % | pubertal males | 95.2 | .0 | .0 | 4.8 | 100 |
|  |  | pre-pubertal males | 3.6 | 78.6 | 14.3 | 3.6 | 100 |
|  |  | pubertal females | .0 | .0 | 100.0 | 0 | 100 |
|  |  | pre-pubertal females | 14.3 | .0 | .0 | 85.7 | 100 |
| **Cross-validated** | count | pubertal males | 3 | 8 | 4 | 6 | 21 |
|  |  | pre-pubertal males | 5 | 3 | 11 | 9 | 28 |
|  |  | pubertal females | 3 | 10 | 7 | 6 | 26 |
|  |  | pre-pubertal females | 6 | 2 | 4 | 2 | 14 |
|  | % | pubertal males | 14.3 | 38.1 | 19.0 | 28.6 | 100 |
|  |  | pre-pubertal males | 17.9 | 10.7 | 39.3 | 32.1 | 100 |
|  |  | pubertal females | 11.5 | 38.5 | 26.9 | 23.1 | 100 |
|  |  | pre-pubertal females | 42.9 | 14.3 | 28.6 | 14.3 | 100 |

**Table S5.** KEGGs biomarkers in males and females.

| pathway | description | 1: mean rel. freq. (%) | 1: std. dev. (%) | 2: mean rel. freq. (%) | 2: std. dev. (%) | p-values |
| --- | --- | --- | --- | --- | --- | --- |
| ANAEROFRUCAT-PWY | homolactic fermentation | 0.54 | 0.15 | 0.60 | 0.09 | 0.01 |
| COBALSYN-PWY | adenosylcobalamin salvage from cobinamide I | 0.65 | 0.10 | 0.60 | 0.09 | 0.02 |
| DTDPRHAMSYN-PWY | dTDP-L-rhamnose biosynthesis I | 0.64 | 0.17 | 0.72 | 0.12 | 0.03 |
| FASYN-ELONG-PWY | fatty acid elongation -- saturated | 0.57 | 0.22 | 0.65 | 0.15 | 0.05 |
| FERMENTATION-PWY | mixed acid fermentation | 0.36 | 0.12 | 0.42 | 0.11 | 0.02 |
| FUCCAT-PWY | fucose degradation | 0.21 | 0.21 | 0.11 | 0.10 | 0.01 |
| GLCMANNANAUT-PWY | superpathway of N-acetylglucosamine, N-acetylmannosamine and N-acetylneuraminate degradation | 0.35 | 0.16 | 0.25 | 0.11 | 0.00 |
| GLUCONEO-PWY | gluconeogenesis I | 0.66 | 0.09 | 0.70 | 0.06 | 0.01 |
| GLYCOLYSIS | glycolysis I (from glucose 6-phosphate) | 0.62 | 0.16 | 0.70 | 0.09 | 0.01 |
| GLYCOLYSIS-E-D | superpathway of glycolysis and Entner-Doudoroff | 0.27 | 0.11 | 0.33 | 0.09 | 0.01 |
| HEXITOLDEGSUPER-PWY | superpathway of hexitol degradation (bacteria) | 0.17 | 0.12 | 0.10 | 0.09 | 0.01 |
| NAGLIPASYN-PWY | lipid IVA biosynthesis | 0.32 | 0.13 | 0.39 | 0.12 | 0.01 |
| NONOXIPENT-PWY | pentose phosphate pathway (non-oxidative branch) | 1.12 | 0.19 | 1.03 | 0.10 | 0.01 |
| P221-PWY | octane oxidation | 0.03 | 0.05 | 0.01 | 0.01 | 0.05 |
| P461-PWY | hexitol fermentation to lactate, formate, ethanol and acetate | 0.23 | 0.27 | 0.10 | 0.12 | 0.00 |
| POLYISOPRENSYN-PWY | polyisoprenoid biosynthesis (E. coli) | 0.44 | 0.14 | 0.51 | 0.10 | 0.00 |
| PRPP-PWY | superpathway of histidine, purine, and pyrimidine biosynthesis | 0.30 | 0.12 | 0.37 | 0.11 | 0.00 |
| PWY-4984 | urea cycle | 0.08 | 0.07 | 0.11 | 0.08 | 0.02 |
| PWY-5154 | L-arginine biosynthesis III (via N-acetyl-L-citrulline) | 0.34 | 0.14 | 0.40 | 0.12 | 0.02 |
| PWY-5188 | tetrapyrrole biosynthesis I (from glutamate) | 0.36 | 0.14 | 0.30 | 0.11 | 0.02 |
| PWY-5189 | tetrapyrrole biosynthesis II (from glycine) | 0.35 | 0.15 | 0.28 | 0.11 | 0.02 |
| PWY-5484 | glycolysis II (from fructose 6-phosphate) | 0.51 | 0.17 | 0.61 | 0.12 | 0.00 |
| PWY-5509 | adenosylcobalamin biosynthesis from cobyrinate a,c-diamide I | 0.63 | 0.10 | 0.57 | 0.10 | 0.02 |
| PWY-621 | sucrose degradation III (sucrose invertase) | 0.61 | 0.20 | 0.49 | 0.16 | 0.00 |
| PWY-6269 | adenosylcobalamin salvage from cobinamide II | 0.63 | 0.10 | 0.58 | 0.10 | 0.02 |
| PWY-6317 | galactose degradation I (Leloir pathway) | 0.63 | 0.13 | 0.53 | 0.11 | 0.00 |
| PWY-6383 | mono-trans, poly-cis decaprenyl phosphate biosynthesis | 0.00 | 0.00 | 0.00 | 0.00 | 0.03 |
| PWY-6467 | Kdo transfer to lipid IVA III (Chlamydia) | 0.26 | 0.11 | 0.32 | 0.11 | 0.01 |
| PWY-6470 | peptidoglycan biosynthesis V (&beta;-lactam resistance) | 0.02 | 0.03 | 0.01 | 0.01 | 0.04 |
| PWY-6572 | chondroitin sulfate degradation I (bacterial) | 0.05 | 0.09 | 0.09 | 0.08 | 0.01 |
| PWY-6609 | adenine and adenosine salvage III | 0.68 | 0.09 | 0.63 | 0.11 | 0.02 |
| PWY-6895 | superpathway of thiamin diphosphate biosynthesis II | 0.34 | 0.12 | 0.39 | 0.08 | 0.02 |
| PWY-7184 | pyrimidine deoxyribonucleotides de novo biosynthesis I | 0.34 | 0.09 | 0.38 | 0.07 | 0.02 |
| PWY-7187 | pyrimidine deoxyribonucleotides de novo biosynthesis II | 0.41 | 0.09 | 0.45 | 0.06 | 0.01 |
| PWY-7199 | pyrimidine deoxyribonucleosides salvage | 0.42 | 0.12 | 0.47 | 0.10 | 0.04 |
| PWY-7200 | superpathway of pyrimidine deoxyribonucleoside salvage | 0.35 | 0.09 | 0.39 | 0.06 | 0.02 |
| PWY-7211 | superpathway of pyrimidine deoxyribonucleotides de novo biosynthesis | 0.29 | 0.11 | 0.36 | 0.10 | 0.00 |
| PWY-7234 | inosine-5'-phosphate biosynthesis III | 0.16 | 0.10 | 0.21 | 0.12 | 0.02 |
| PWY-7332 | superpathway of UDP-N-acetylglucosamine-derived O-antigen building blocks biosynthesis | 0.02 | 0.02 | 0.04 | 0.05 | 0.03 |
| PWY-7371 | 1,4-dihydroxy-6-naphthoate biosynthesis II | 0.14 | 0.23 | 0.06 | 0.08 | 0.03 |
| PWY-7377 | cob(II)yrinate a,c-diamide biosynthesis I (early cobalt insertion) | 0.26 | 0.19 | 0.15 | 0.14 | 0.00 |
| PWY-7456 | mannan degradation | 0.17 | 0.12 | 0.24 | 0.12 | 0.01 |
| PWY0-1296 | purine ribonucleosides degradation | 0.51 | 0.12 | 0.40 | 0.09 | 0.00 |
| PWY0-1298 | superpathway of pyrimidine deoxyribonucleosides degradation | 0.48 | 0.15 | 0.38 | 0.09 | 0.00 |
| PWY0-1586 | peptidoglycan maturation (meso-diaminopimelate containing) | 0.62 | 0.34 | 0.47 | 0.19 | 0.01 |
| PWY0-166 | superpathway of pyrimidine deoxyribonucleotides de novo biosynthesis (E. coli) | 0.39 | 0.09 | 0.44 | 0.05 | 0.00 |
| PWY0-845 | superpathway of pyridoxal 5'-phosphate biosynthesis and salvage | 0.15 | 0.10 | 0.20 | 0.11 | 0.02 |
| PYRIDOXSYN-PWY | pyridoxal 5'-phosphate biosynthesis I | 0.12 | 0.08 | 0.16 | 0.09 | 0.01 |

1: male subjects; 2: female subjects

**Table S6.** KEGGs biomarkers in pubertal males and pubertal females.

| pathway | description | 1: mean rel. freq. (%) | 1: std. dev. (%) | 2: mean rel. freq. (%) | 2: std. dev. (%) | p-values |
| --- | --- | --- | --- | --- | --- | --- |
| ANAEROFRUCAT-PWY | homolactic fermentation | 0.49 | 0.15 | 0.61 | 0.07 | 0.00 |
| ARO-PWY | chorismate biosynthesis I | 0.82 | 0.10 | 0.76 | 0.09 | 0.04 |
| COBALSYN-PWY | adenosylcobalamin salvage from cobinamide I | 0.68 | 0.11 | 0.60 | 0.10 | 0.01 |
| CODH-PWY | reductive acetyl coenzyme A pathway | 0.02 | 0.02 | 0.05 | 0.07 | 0.03 |
| COLANSYN-PWY | colanic acid building blocks biosynthesis | 0.31 | 0.09 | 0.37 | 0.09 | 0.04 |
| COMPLETE-ARO-PWY | superpathway of aromatic amino acid biosynthesis | 0.87 | 0.11 | 0.79 | 0.09 | 0.03 |
| FERMENTATION-PWY | mixed acid fermentation | 0.37 | 0.12 | 0.45 | 0.09 | 0.02 |
| FUCCAT-PWY | fucose degradation | 0.25 | 0.21 | 0.13 | 0.09 | 0.03 |
| GLCMANNANAUT-PWY | superpathway of N-acetylglucosamine, N-acetylmannosamine and N-acetylneuraminate degradation | 0.38 | 0.16 | 0.28 | 0.11 | 0.02 |
| GLYCOLYSIS | glycolysis I (from glucose 6-phosphate) | 0.57 | 0.15 | 0.70 | 0.08 | 0.00 |
| GLYCOLYSIS-E-D | superpathway of glycolysis and Entner-Doudoroff | 0.24 | 0.11 | 0.34 | 0.07 | 0.00 |
| HISDEG-PWY | L-histidine degradation I | 0.13 | 0.12 | 0.22 | 0.12 | 0.02 |
| NAGLIPASYN-PWY | lipid IVA biosynthesis | 0.27 | 0.12 | 0.36 | 0.11 | 0.02 |
| NONOXIPENT-PWY | pentose phosphate pathway (non-oxidative branch) | 1.18 | 0.20 | 1.03 | 0.12 | 0.01 |
| P441-PWY | superpathway of N-acetylneuraminate degradation | 0.32 | 0.12 | 0.39 | 0.07 | 0.03 |
| P461-PWY | hexitol fermentation to lactate, formate, ethanol and acetate | 0.28 | 0.27 | 0.12 | 0.12 | 0.02 |
| POLYISOPRENSYN-PWY | polyisoprenoid biosynthesis (E. coli) | 0.40 | 0.15 | 0.49 | 0.10 | 0.02 |
| PRPP-PWY | superpathway of histidine, purine, and pyrimidine biosynthesis | 0.25 | 0.11 | 0.37 | 0.11 | 0.00 |
| PWY-4984 | urea cycle | 0.04 | 0.03 | 0.09 | 0.07 | 0.01 |
| PWY-5484 | glycolysis II (from fructose 6-phosphate) | 0.44 | 0.15 | 0.61 | 0.09 | 0.00 |
| PWY-5509 | adenosylcobalamin biosynthesis from cobyrinate a,c-diamide I | 0.66 | 0.11 | 0.58 | 0.11 | 0.02 |
| PWY-5676 | acetyl-CoA fermentation to butanoate II | 0.09 | 0.06 | 0.14 | 0.10 | 0.04 |
| PWY-5913 | TCA cycle VI (obligate autotrophs) | 0.23 | 0.14 | 0.32 | 0.14 | 0.05 |
| PWY-6163 | chorismate biosynthesis from 3-dehydroquinate | 0.82 | 0.10 | 0.76 | 0.09 | 0.03 |
| PWY-6165 | chorismate biosynthesis II (archaea) | 0.00 | 0.00 | 0.00 | 0.00 | 0.05 |
| PWY-621 | sucrose degradation III (sucrose invertase) | 0.70 | 0.19 | 0.54 | 0.13 | 0.00 |
| PWY-6269 | adenosylcobalamin salvage from cobinamide II | 0.66 | 0.11 | 0.58 | 0.11 | 0.02 |
| PWY-6317 | galactose degradation I (Leloir pathway) | 0.70 | 0.11 | 0.57 | 0.09 | 0.00 |
| PWY-6467 | Kdo transfer to lipid IVA III (Chlamydia) | 0.22 | 0.10 | 0.29 | 0.10 | 0.01 |
| PWY-6572 | chondroitin sulfate degradation I (bacterial) | 0.02 | 0.02 | 0.11 | 0.10 | 0.00 |
| PWY-6609 | adenine and adenosine salvage III | 0.72 | 0.10 | 0.63 | 0.12 | 0.01 |
| PWY-6891 | thiazole biosynthesis II (Bacillus) | 0.34 | 0.15 | 0.24 | 0.11 | 0.02 |
| PWY-6892 | thiazole biosynthesis I (E. coli) | 0.68 | 0.13 | 0.59 | 0.09 | 0.01 |
| PWY-6901 | superpathway of glucose and xylose degradation | 0.33 | 0.13 | 0.40 | 0.10 | 0.04 |
| PWY-6906 | chitin derivatives degradation | 0.00 | 0.00 | 0.00 | 0.00 | 0.03 |
| PWY-7090 | UDP-2,3-diacetamido-2,3-dideoxy-&alpha;-D-mannuronate biosynthesis | 0.00 | 0.00 | 0.01 | 0.01 | 0.01 |
| PWY-7184 | pyrimidine deoxyribonucleotides de novo biosynthesis I | 0.30 | 0.11 | 0.37 | 0.07 | 0.02 |
| PWY-7187 | pyrimidine deoxyribonucleotides de novo biosynthesis II | 0.37 | 0.10 | 0.44 | 0.05 | 0.01 |
| PWY-7211 | superpathway of pyrimidine deoxyribonucleotides de novo biosynthesis | 0.26 | 0.11 | 0.34 | 0.09 | 0.01 |
| PWY-7220 | adenosine deoxyribonucleotides de novo biosynthesis II | 0.64 | 0.17 | 0.78 | 0.14 | 0.01 |
| PWY-7222 | guanosine deoxyribonucleotides de novo biosynthesis II | 0.64 | 0.17 | 0.78 | 0.14 | 0.01 |
| PWY-7234 | inosine-5'-phosphate biosynthesis III | 0.12 | 0.08 | 0.22 | 0.11 | 0.00 |
| PWY-7328 | superpathway of UDP-glucose-derived O-antigen building blocks biosynthesis | 0.09 | 0.07 | 0.15 | 0.08 | 0.01 |
| PWY-7332 | superpathway of UDP-N-acetylglucosamine-derived O-antigen building blocks biosynthesis | 0.01 | 0.01 | 0.04 | 0.04 | 0.00 |
| PWY-7377 | cob(II)yrinate a,c-diamide biosynthesis I (early cobalt insertion) | 0.28 | 0.18 | 0.16 | 0.13 | 0.02 |
| PWY-7392 | taxadiene biosynthesis (engineered) | 0.17 | 0.10 | 0.27 | 0.12 | 0.01 |
| PWY0-1241 | ADP-L-glycero-&beta;-D-manno-heptose biosynthesis | 0.08 | 0.05 | 0.14 | 0.07 | 0.00 |
| PWY0-1261 | anhydromuropeptides recycling | 0.22 | 0.12 | 0.36 | 0.16 | 0.00 |
| PWY0-1296 | purine ribonucleosides degradation | 0.57 | 0.11 | 0.44 | 0.07 | 0.00 |
| PWY0-1298 | superpathway of pyrimidine deoxyribonucleosides degradation | 0.54 | 0.12 | 0.41 | 0.08 | 0.00 |
| PWY0-1586 | peptidoglycan maturation (meso-diaminopimelate containing) | 0.69 | 0.34 | 0.49 | 0.14 | 0.02 |
| PWY0-166 | superpathway of pyrimidine deoxyribonucleotides de novo biosynthesis (E. coli) | 0.36 | 0.10 | 0.43 | 0.05 | 0.01 |
| PWY0-781 | aspartate superpathway | 0.25 | 0.10 | 0.31 | 0.09 | 0.04 |
| PWY0-845 | superpathway of pyridoxal 5'-phosphate biosynthesis and salvage | 0.11 | 0.09 | 0.20 | 0.10 | 0.00 |
| PYRIDNUCSYN-PWY | NAD biosynthesis I (from aspartate) | 0.63 | 0.11 | 0.56 | 0.11 | 0.04 |
| PYRIDOXSYN-PWY | pyridoxal 5'-phosphate biosynthesis I | 0.08 | 0.07 | 0.16 | 0.09 | 0.00 |
| TRPSYN-PWY | L-tryptophan biosynthesis | 0.69 | 0.13 | 0.59 | 0.10 | 0.00 |

1: pubertal male subjects; 2: pubertal female subjects

**Table S7.** KEGGs biomarkers in pre-pubertal males and pre-pubertal females.

| pathway | description | 1: mean rel. freq. (%) | 1: std. dev. (%) | 2: mean rel. freq. (%) | 2: std. dev. (%) | p-values |
| --- | --- | --- | --- | --- | --- | --- |
| 1CMET2-PWY | N10-formyl-tetrahydrofolate biosynthesis | 0.65 | 0.09 | 0.71 | 0.03 | 0.00 |
| ANAGLYCOLYSIS-PWY | glycolysis III (from glucose) | 0.80 | 0.11 | 0.86 | 0.05 | 0.02 |
| FUC-RHAMCAT-PWY | superpathway of fucose and rhamnose degradation | 0.17 | 0.09 | 0.11 | 0.05 | 0.01 |
| GLCMANNANAUT-PWY | superpathway of N-acetylglucosamine, N-acetylmannosamine and N-acetylneuraminate degradation | 0.36 | 0.15 | 0.27 | 0.10 | 0.03 |
| GLUCONEO-PWY | gluconeogenesis I | 0.67 | 0.08 | 0.72 | 0.03 | 0.00 |
| HEMESYN2-PWY | heme biosynthesis II (anaerobic) | 0.08 | 0.07 | 0.04 | 0.03 | 0.03 |
| PWY-5005 | biotin biosynthesis II | 0.12 | 0.09 | 0.07 | 0.06 | 0.05 |
| PWY-5188 | tetrapyrrole biosynthesis I (from glutamate) | 0.37 | 0.13 | 0.27 | 0.11 | 0.02 |
| PWY-5189 | tetrapyrrole biosynthesis II (from glycine) | 0.35 | 0.13 | 0.26 | 0.10 | 0.02 |
| PWY-6895 | superpathway of thiamin diphosphate biosynthesis II | 0.31 | 0.13 | 0.39 | 0.10 | 0.03 |
| PWY-7199 | pyrimidine deoxyribonucleosides salvage | 0.43 | 0.11 | 0.50 | 0.08 | 0.03 |
| PWY-7211 | superpathway of pyrimidine deoxyribonucleotides de novo biosynthesis | 0.27 | 0.08 | 0.33 | 0.09 | 0.03 |
| PWY-7456 | mannan degradation | 0.16 | 0.11 | 0.25 | 0.10 | 0.01 |
| PWY0-1296 | purine ribonucleosides degradation | 0.55 | 0.11 | 0.44 | 0.11 | 0.01 |
| PYRIDNUCSAL-PWY | NAD salvage pathway I | 0.59 | 0.10 | 0.67 | 0.04 | 0.00 |

1: pre-pubertal male subjects; 2: pre-pubertal female subjects
